# Correlation between anti-PD-L1 tumor concentrations and tumor-specific and nonspecific biomarkers in a melanoma mouse model

**DOI:** 10.18632/oncotarget.12727

**Published:** 2016-10-18

**Authors:** Ana M. Contreras, María Merino, Marcos Vasquez, Iñaki F. Trocóniz, Pedro Berraondo, María J. Garrido

**Affiliations:** ^1^ School of Pharmacy, Department of Pharmacy and Pharmaceutical Technology, University of Navarra, 31008 Pamplona, Spain; ^2^ Program of Immunology and Immunotherapy, Center for Applied Medical Research (CIMA), Navarra Institute for Health Research (IdiSNA), Pamplona, Navarra, 31008, Spain

**Keywords:** immunomodulation, anti-PD-L1 mAb, preclinical study, melanoma model, biomarkers

## Abstract

Blockade of PD-L1 with specific monoclonal antibodies (anti-PD-L1) represents a therapeutic strategy to increase the capability of the immune system to modulate the tumor immune-resistance. The relationship between anti-PD-L1 tumor exposition and anti-tumor effect represents a challenge that has been addressed in this work through the identification of certain biomarkers implicated in the antibody's mechanism of action, using a syngeneic melanoma mouse model. The development of an *in-vitro*/*in-vivo* platform has allowed us to investigate the PD-L1 behavior after its blockage with anti-PD-L1 at cellular level and in animals. *In-vitro* studies showed that the complex PD-L1/anti-PD-L1 was retained mainly at the cell surface. The antibody concentration and time exposure affected directly the recycling or ligand turnover. *In-vivo* studies showed that anti-PD-L1 was therapeutically active at all stage of the disease, with a rapid onset, a low but durable efficacy and non-relevant toxic effect. This efficacy measured as tumor shrinkage correlated with tumor-specific infiltrating lymphocytes (TILs), which increased as antibody tumor concentrations increased. Both, TILS and antibody concentrations followed similar kinetic patterns, justifying the observed anti-PD-L1 rapid onset. Interestingly, peripheral lymphocytes (PBLs) behave as infiltrating lymphocytes, suggesting that these PBLs might be considered as a possible biomarker for antibody activity.

## INTRODUCTION

Immune-checkpoint targeting represents a new therapeutic strategy in oncology. Programmed death-1 (PD-1) receptor and its ligand (PD-L1) is one of the axes able to up- or down regulate the immune response against tumors. PD-1 is expressed by activated T-cells in peripheral tissues and tumor microenvironment. PD-L1 is upregulated on the surface of many types of solid tumor cells, although its expression level is heterogeneous and shows an active dynamics. Therefore, when PD-1 engages PD-L1, it transduces an inhibitory signal to T-cells limiting their expansion and effector functions, leading to tumor immunoresistance. Based on that, the PD-1/PD-L1 blockade represents a therapeutic approach able to abolish this negative regulatory mechanism of T-cell activation and thereby promoting the antitumor immunity [1]. In several animal models, a relationship between a high PD-L1 expression in tumors and T-cells inhibition limiting the antitumor response has been established [2, 3].

Currently, monoclonal antibodies (mAbs) represent the main approach to block the activity of these PD-L1 and PD-1 immune checkpoints. Regarding PD-1, Pembrolizumab and Nivolumab, both, for melanoma and locally advanced or metastatic squamous non-small cell lung cancer (NSCLC), have been recently approved for clinical use [4]. Meanwhile, Atezolizumab, an anti-PD-L1 has been approved in this year by FDA for advanced bladder cancer [5]. However, many others anti-PD-L1 mAbs are still involved in several clinical trials enrolling patients with different types of solid tumors. Note that results from these trials reported that only 21–17% of patients presented an objective and durable response, which represents a low efficacy of anti-PD-L1 treatment. Although, it is worthy to remark its low toxicity [6, 7, 8]. In order to improve this therapeutic efficiency, several approaches, based mainly on different combinations of anti-PD-L1 with other agents, including the combination with anti-PD-1, are being tested. However, PD-L1 activity as well as anti-PD-L1 immune-induced mechanism remain yet unexplored in detail [9]. In that sense, it is known that PD-L1 is involved in two types of immune resistance mechanisms that can co-exist: i) intrinsic, referred to PD-L1 expression induced by certain signalling pathways, AKT and STAT3, in cancer cells, and ii) adaptive, referred to the auto-induction of this ligand by the presence of some cytokines such as IFN-γ [4]. Additionally, in recent years, this ligand has been described as the best biomarker in order to treat patients classified as PD-L1+ with anti-PD-1, as first line therapy [10]. However, the question about how PD-L1 expression may be modulated by anti-PD-L1 triggering antitumor response arouses great interest among researchers, at this time.

The use of animal models is a very useful tool to investigate the relationship between pharmacokinetics (PK) and pharmacodynamics (PD) of anti-PD-L1. PK-PD models, widely applied to many conventional antitumor agents, are able to describe and predict tumor dynamics according to plasma or tumor drug concentrations [11]. Thus, based on these models, it is expected that anti-PD-L1 tumor levels may control PD-L1 availability and then, induce therapeutic response, but this is not really established yet. However, the objective of this work is to explore in a qualitative way the anti-PD-L1 tumor kinetics as well as the expression of certain biomarkers associated with the immunomodulation induced by this antibody, in order to provide information about the mechanisms of anti-PD-L1 to attain antitumor effect. To address this, an *in-vitro/in-vivo* platform has been developed.

## RESULTS

### *In-vitro* studies

#### Anti-PD-L1 binds specifically to PD-L1 and the complex remains mainly at the surface of the cells

PD-L1 expression characterized by flow cytometry analysis showed that approx. 100% of B16-OVA cells were PD-L1+.

**Figure 1:**
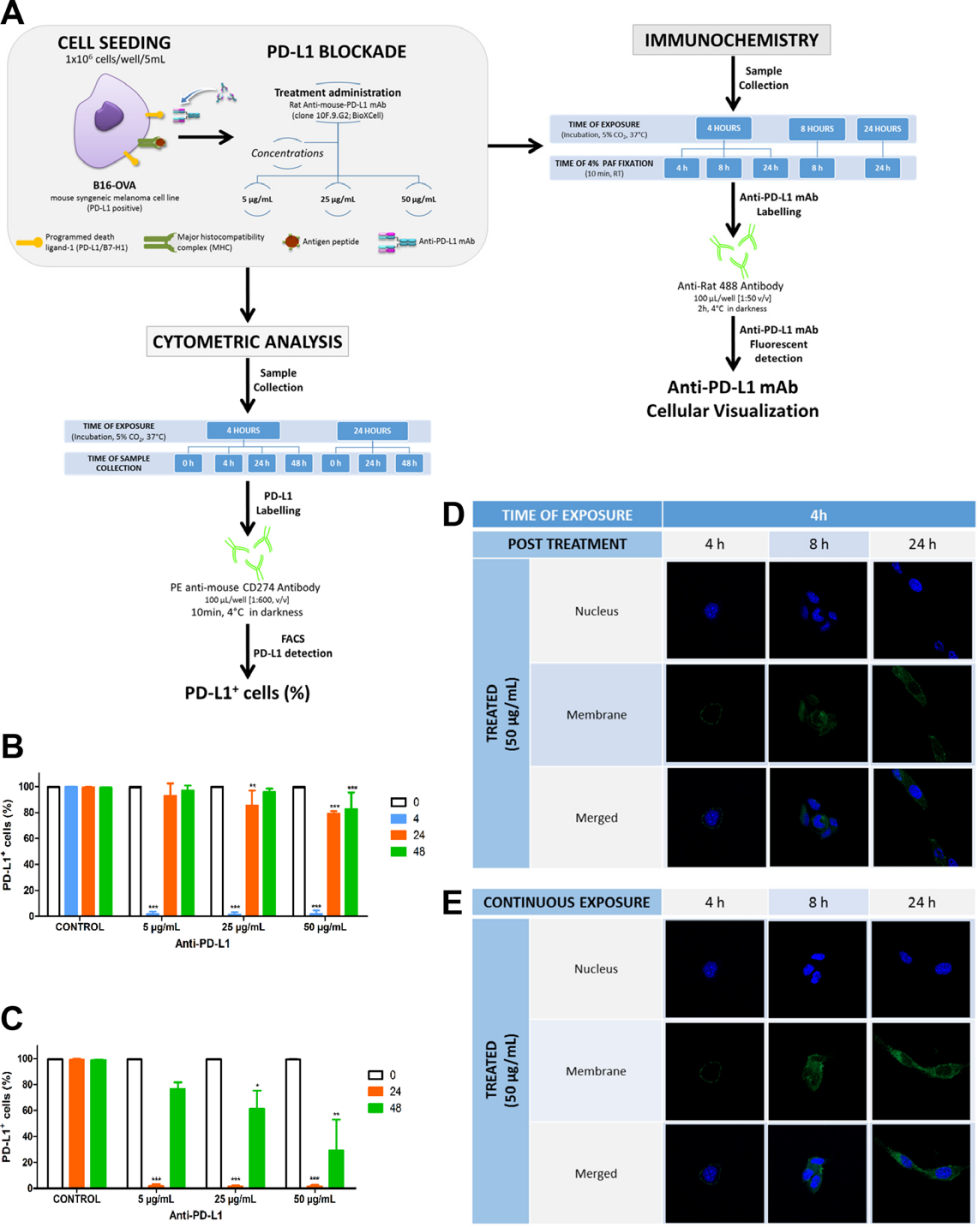
Effect of different anti-PD-L1 concentrations on PD-L1 availability and cells imaged by confocal microscopy to visualize complex localization (**A**) *In-vitro* design: Cells seeded at density of 1 × 10^6^ cells/well and exposure for 4 or 24 h at 5, 25 and 50 μg/mL of anti-PD-L were analysed by flow cytometer to quantify the PD-L1 availability over 48 h; at the same time, cells treated with 50 μg/mL for different times of exposure were fixed, stained and analyzed by con-focal microscopy. (**B** and **C**) represent the percentage of cells expressing PD-L1 over 48 h in control and after 4 h and 24 h exposure to three different antibody concentrations. Statistical differences were calculated between control and treatments and across times for each treatment (***p < 0.001; **p < 0.01; *p > 0.5). Data represent the mean ± SD of three independent experiments. (**D** and **E**) Immunohistochemical images show the location of PD-L1/ mAb in culture cells (green). Cells, incubated with 50 μg/mL anti-PD-L1 mAb at 37°C, were fluorescently labeled with mAb against anti-PD-L1 and with dapi for nuclei; upper panels correspond to cells treated for 4 h and visualized just after treatment, 4 h, and during 24 h post-treatment (panels D), and cells exposed continuously to the treatment for 24 h (panels E).

Figure 1, panels B and C, shows a significant reduction of ligand availability at the cell surface after four or twenty-four hours exposure to 5, 25 or 50 μg/mL of anti-PD-L1. Ligand turnover or recycling was dependent on the time exposure to treatment as well as the antibody concentration. Thus, PD-L1 availability at 24 h post-treatment was recovered in almost 100% of cells exposed to 5 μg/mL anti-PD-L1 for 4 h (Figure 1B), whereas the same concentration after 24 h incubation led to a recovery of 80% (Figure 1C). At the same time, the ligand availability in cells treated with 50 μg/mL of anti-PD-L1 for 4 h, was recovered in 80% compared to 40% after 24 h of exposure. This finding proves that these variables, time exposure and antibody concentration, may be very relevant for controlling ligand availability. Nevertheless, because PD-L1 recovery took longer after the exposure to the highest anti-PD-L1 concentration and time exposure, this may suggest the ligand internalization in addition to the ligand blockade at the cell surface. To explore these blocking/recycling processes of PD-L1, attached cells and in suspension were treated for 4 h with 25 μg/mL anti-PD-L1 at 4°C and 37°C, respectively. Table 1 shows that PD-L1 could not be detected at any condition, demonstrating that anti-PD-L1 specifically bound to PD-L1 expressing tumor cells, blocking its detection at the surface. Images using confocal microscopy allowed us to confirm this point. Figure 1 shows that after 4 h exposure to 50 μg/mL anti-PD-L1 (Figure 1D), the antibody was mainly bound to cell membrane. However as the time exposure to treatment increases to 24 h (Figure 1E), the uptake increases too, being more evident the intensity of the labelling. In the same way, the signal becomes weaker at 24 h after 4 h exposure to antibody (Figure 1D). Therefore, the dynamic of PD-L1/anti-PD-L1 interaction is compatible with a mechanism of ligand association with the antibody at the cell surface followed by the uptake/ internalization of the complex. Being the former the main mechanism involved.

**Table 1:** Cells expressing PD-L1 (%) in control and treated with 25 μg/mL anti-PD-L1 for 4h

Exposure conditions	Culture conditions	Time(h)	PD-L1^+^ cell (%)	
			Control	25μg/mL
**4°C**	Attached	0	92.8± 0.47	n.a.
		4	95.1 ± 0.06	0.26 ± 0.05
	In suspension	0	92.1 ± 0.10	n.a.
		4	91.4 ± 1.80	0.29 ± 0.09
**37°C**	Attached	0	92.8 ± 0.47	n.a.
		4	90.6 ± 0.40	0.3 ± 0.04

Data represent the mean ± SD of three independent experiments.

### *In-vivo* experiments

#### Influence of initial tumor size on anti-PD-L1 activity

**Figure 2:**
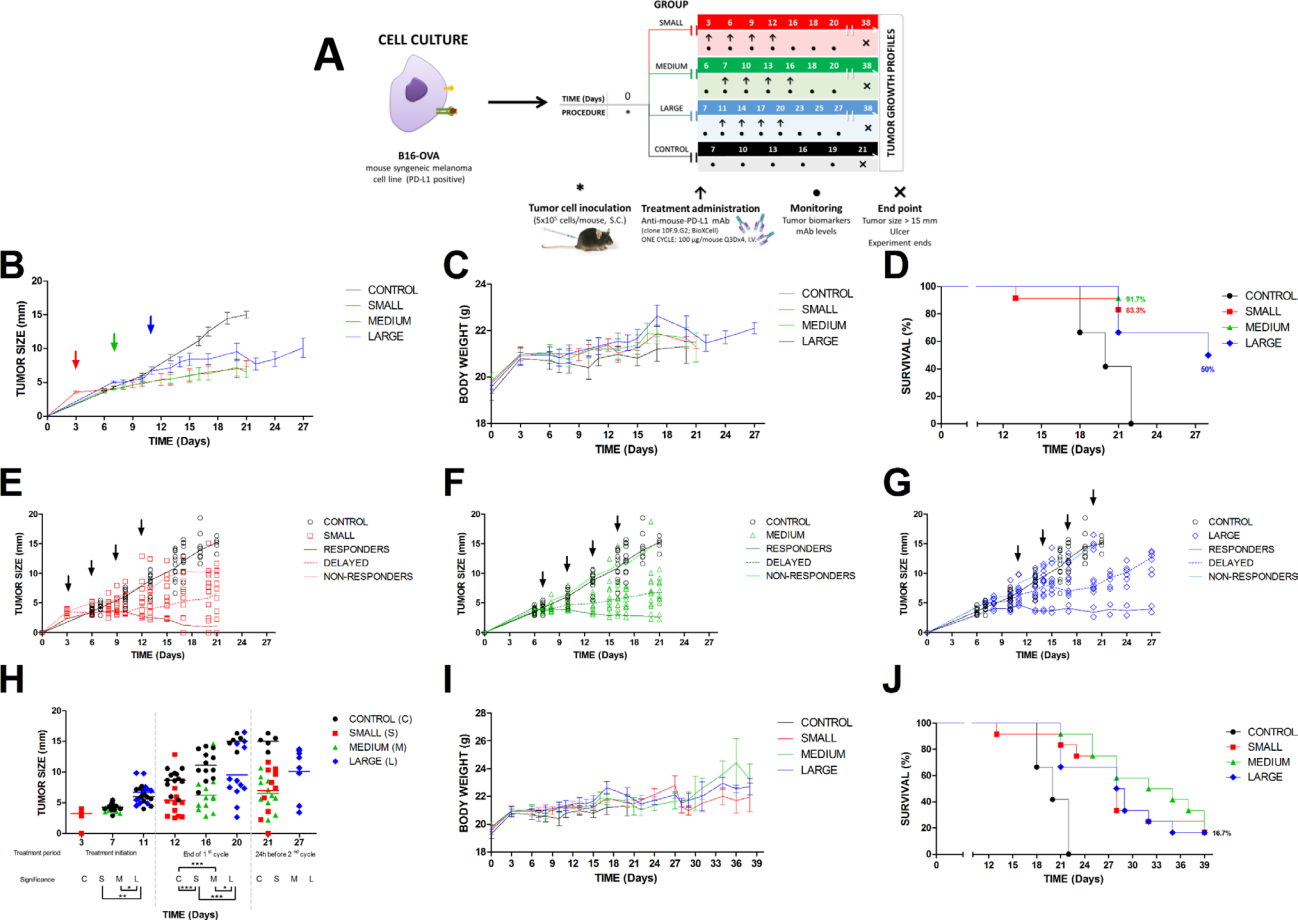
Pharmacodynamic of anti-PD-L1 administered to tumor bearing mice (**A**) experimental design for one cycle of treatment. Data are represented for control and treated groups according to different stages of the disease, small, medium and large tumor size; (**B**) Time profile of the mean tumor growth. Arrows show the starting time of the treatment at day 3, 7 and 11, respectively; (**C**) Time profile of the mean body weight; (**D**) Kaplan-Meier curve representing the results from the first cycle of treatment; (**E**–**G**) individual tumor growth kinetics. Symbols represent observations, solid lines mean tendency of control (black), responders and non-responders (different colours) and coloured dashed lines animals with a delayed effect. Arrows show the four doses corresponding to first cycle; (**H**) Individual tumor size represented by circles for control and treated groups (small, medium and large) just at the day of first and fourth dose administration and 24 h before starting the second cycle of treatment. Statistical differences were calculated across groups (*P < 0.05; **P < 0.01; ***P < 0.001). Overall data for the two cycles of treatment are shown in panels: (**I**) time profile of the mean body weight, and (**J**) Kaplan-Meier curve for the complete study.

In order to simulate the progression of the disease in a preclinical mouse model, different initial tumor sizes, corresponding to different times of tumor growth have been used to assess the antitumor effect of the antibody (Figure 2A) [12]. The efficacy of anti-PD-L1 in controlling the tumor growth was not influenced by this experimental approach. Thus, as is observed in Table 2, the tumor size in all treated groups was incremented in approx. 2 mm at the end of the first cycle comparing to their respective initial sizes. Therefore, this effect triggered by the anti-PD-L1 administration was independent of the tumor size at the beginning of the first dose (Figure 2E–2G), suggesting that this status of the disease (small, medium or large) did not impact significantly in the antibody activity. In fact, in the three groups, 2 mice/group attained total tumor regression with the first cycle of treatment, one mouse/group at the end of the fourth dose and the second mouse/group during the washout period before starting the second cycle. Additionally, this second cycle did not provide any other new tumor regressions (Table 2). These results are also graphically represented in detail in Figure 2. Panel B shows that the onset was rapidly observed just after the first dose administration. At this time, tumor growth profiles started to become flatter in comparison with control group. This difference between treated and control groups was higher after the third antibody administration, suggesting that at least, three doses were necessary to observe an antitumor effect. Nevertheless, the optimization of the dosing regimen was not addressed in this work. Panels E, F and G in Figure 2 represent individual time profiles for each group. In all the cases, it was evident the presence of responders and non-responders. Responders were classified as those mice presenting a delay in the tumor growth (delayed effect) and mice that achieved a total regression. In the case of non-responders, they behave similar to control group. Based on this, the mean antitumor effect value was not very representative due to the high interindividual variability observed in each group. Even though, statistical differences could be found across these groups after the last dose (Figure 2H).

**Table 2:** Summary of the antitumor effect and the toxicity caused by anti-PD-L1 administration to mice at different days after B16-OVA cells inoculation

CYCLE OF TREATMENT (Q3D × 4 doses.)		GROUPS				STS	MTS	LTS
**1st**	1st dose.		Day			3	7	11
		Tumor size (mm ± SEM)		Treated		3.26 ± 0.31	3.98 ± 0.13	6.71 ± 0.51
				Control		—	4.27 ± 0.19	6.01 ± 0.32
	4th dose.		Day			12	16	20
		Tumor size (mm ± SEM)		Treated		5.40 ± 0.88	6.26 ± 0.92	9.52 ± 1.29
				Control		8.70 ± 0.46	11.14 ± 0.69	15.02 ± 0.51
		**Evaluation** (N° mice)		**Responders**		***1***	***1***	***1***
				Delayed tumor growth		4	4	3
				Non-responders		7	7	7
			Deaths	Treated		0	0	4
				Control		7		
**2nd**	Not-receiving		Day			21	21	27
		**Evaluation** (N° mice)		**Total regression**		2*	2*	2*
				Deaths	Treated	2	1	2
	1^st^ dose.		Day			22	22	28
		Tumor size (mm ± SEM)		Treated		9.21 ± 1.15	7.65 ± 0.60	11.55 ± 0.66
				Control		15.54 ± 0.36.		n.a
	Experiment ends (Day 39)	**Evaluation** (N° mice)		Deaths	Treated	8	9	4

STS, Small initial tumor size; MTS, Medium initial tumor size; LTS, Large initial tumor size. —, No data available.

* Total number of mice; n.a., Non-applicable.

Tumor size and death were recorded for each group (*n* = 12/group), three treated and control, throughout 40 days, corresponding to the two cycles of treatment. Therapeutic evaluation is reported after the first and fourth dose of the first cycle and for the first dose, in the case of the second cycle. Note that at day 20, five control mice died compared to the four non-responders of the large group. None of control mice survived at day 28. Tumor size is expressed as Mean ± SEM.

Survival curve, represented in Figure 2J, showed that anti-PD-L1 was able to provide the same therapeutic outcome with independence of initial tumor size. It can be observed that 17% of mice kept alive and cured at the end of the study. This means that two mice per group showed an extended survival, remaining in the study until day 40 with total tumor regression and without any associated toxicity receiving only one cycle of treatment. In this work, toxic effect was evaluated by monitoring the body weight, which did not suffered significant changes over time (Figure 2C and 2I). Although some animals died, this could not be directly attributable to treatment. Figure 2D shows that the higher number of deaths occurred in the large group. At the end of the first cycle only 50% of individuals remained in the study for the large group compared to > 80% in medium and small group, respectively. This type of cancer is very aggressive, affecting mainly to the group with the most advanced disease or large tumor size. Therefore, therapeutic activity of anti-PD-L1 was found in the three groups characterized by a rapid onset, a low but durable efficacy and non-toxic effect.

### Antibody tumor exposure correlated with specific infiltrating lymphocytes

**Figure 3:**
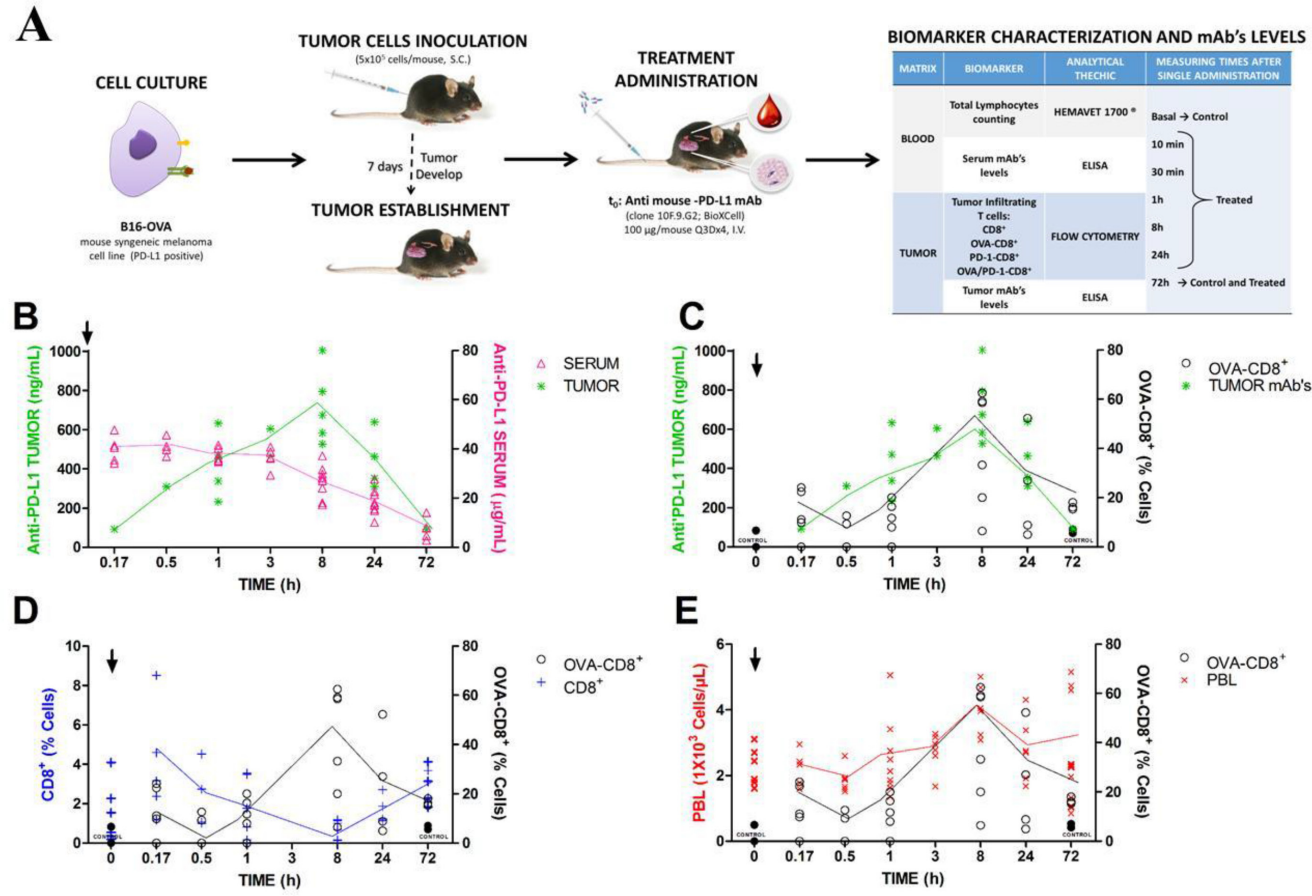
Anti-PD-L1 treatment induces specific biomarkers expression *in-vivo* Observations were individually collected after first mAb dose administration (100 μg/mouse i.v.). (**A**) experimental design; (**B**) time profile of mAb concentrations in serum and tumor, respectively; (**C**) Specific tumor-infiltrating lymphocytes, OVA-CD8+, vs. mAb tumor concentrations throughout 72 h post-dosing; (**D**) non-specific CD8+ vs. specific, OVA-CD8+ and (**E**) Individual time profiles of OVA-CD8+ vs. peripheral lymphocytes over 72 h. Symbols represent experimental data, solid lines the mean tendency, arrows dosing time and in panels C, D and E, black circles correspond to control data.

In this experiment, the medium group was selected for the evaluation of immune effect of anti-PD-L1 because the interindividual variability was lower than in the other two groups. Tumor kinetics shows that anti-PD-L1 rapidly reached tissue target. Thus, serum concentrations decreased as it increased in tumor, achieving the maximum concentration or Cmax at 8 h post-dosing and declining very fast until 72 h, as is represented in Figure 3B. These tumor antibody concentrations could be correlated with the kinetics of tumor specific infiltrating lymphocytes (OVA-CD8+), as is observed in Figure 3C. This finding suggested that tumor anti-PD-L1 levels triggered the effect on the immune system providing an increment of OVA-CD8+ lymphocytes, which were characterized by a rapid and discrete increment at 10 min, 16.85%, declining very fast until 11.98% at 1 h and increasing again at 8 h, 39.98%, after antibody administration. The lymphocytes kinetic tendency could be clearly identified despite the intersubject variability. This heterogeneous behaviour of the immune system may support the different populations observed for the anti-PD-L1 antitumor effect. Moreover, control group did not show any change in OVA-CD8+ levels, which is consistent with the activity of anti-PD-L1 blocking PD-L1 and then, inhibiting the negative T-cell regulation promoted by this ligand. In that sense, CD8+ levels in tumor decreased as OVA-CD8+ increased, justifying the mechanism of antibody to induce the specific immune response provided by specific infiltrating lymphocytes (Figure 3D). To investigate whether this immune activation might be also detected in blood, peripheral lymphocytes levels (PBL) were measured, as is shown in Figure 3E. PBL showed a discrete modulation throughout 72 h post-dosing. Thus, PBL followed a similar time profile to OVA-CD8+, which permitted to suggest a correlation between both cell populations. In addition, no changes in PBL levels in control mice support the relationship between anti-PD-L1 tumor disposition and PBL in blood. Then, early PBL kinetics may represent a possible biomarker of anti-PD-L1 activity.

## DISCUSSION

This work describes the anti-PD-L1 activity and efficacy in a melanoma syngeneic mouse model as well as the PD-L1 dynamics. To address these, an *in-vitro/in-vivo* platform has been developed using B16-OVA cell line. This cell line has been classified as PD-L1+ according to the analysis by flow cytometry (approx. 100% cells), representing a good model to study the anti-PD-L1/PD-L1 coupling mechanism. The *in-vitro* system allowed us to prove that the blockade takes place mainly at the surface of the tumor cells. This was contrary to the mechanism of uptake and internalization of the complex mAb-ligand, proposed for our group based on the most common mechanism described for several mAb targeted to upregulated molecules in cancer cells [13]. No differences in PD-L1 availability after anti-PD-L1 treatments at 37°C and 4°C support an interaction or association mAb/ligand covering the cellular membrane. This could be confirmed by images using confocal microscopy. However, cells treated with higher antibody concentrations and for longer time (approx. 24 h) were able to internalize the complex because this condition led to a downregulation of PD-L1, clearly evidenced in culture cells (Figure 1C). These results are according to those reported by Heskamp *et al.* (2015) [14]. These authors assayed the *in-vitro* binding of the radiolabeled murine anti-PD-L1 in a PD-L1+ human breast cancer cells, MDA-MB-231. After 2, 4 or 24 h of exposure to anti-PD-L1, they reported a double mechanism, association or interaction ligand-antibody and internalization of the complex, being the association the most relevant; same as was observed in the present work. Thus, these authors found that more than 75% of the dose was attached at the surface, while less than 25% was slowly internalized after 24 h of exposure. In that sense, inhibition of the expression resulted to be independent of antibody concentration and time exposure. However, these both variables influenced the re-cycling of the ligand due to the persistent down-regulation of PD-L1 over time. In this line, Chang and co-workers [15] have also reported the internalization of PD-L1 after 30 min antibody treatment, promoting the cessation of Akt phosphorylation signalling cascade. Note that Chang's study was carried out in a very immunogenic sarcoma cell line. Therefore, it is noticed that types of cells lines as well as its PD-L1 expression profile may explain different results. Because the antibody concentration and time exposure are relevant variables, we analyzed both *in-vivo* by measuring the tumor kinetics of the mAb, as well as its efficacy and toxicity in B16-OVA tumor cells bearing mice. Anti-PD-L1 treated mice showed a tumor shrinkage compared to non-treated mice, demonstrating the antitumor capability of this antibody. This activity seemed not to be influenced by the stage of the disease reflected in this work by the three groups with different initial tumor sizes or time of growth. In fact, similar antitumor effect was found across these treated groups. It is particularly interesting to point out this difference with conventional chemotherapy, which becomes more efficient at the initial stages (small tumor size).

On one hand, the heterogeneity in the response led to distinguish three types of populations, responders, non-responders and mice with a delayed response. Similar responses have been found in clinical trials for these type of immune-modulators [6, 16]. In this work, the criteria used for this classification were: responders, no changes in the initial tumor size or < 1 mm tumor growth at the end of the first cycle; non-responders, similar growing profile to control group during the study and delayed response, tumor growth was 50 ± 10% lower than control group during the treatment. In each treated group, only few animals were non-responders and responders. Thus, total tumor regression was found in < 20% of mice, although this response was durable > 40 days. This data is in line with those reported by several authors in preclinical and clinical studies for mAbs targeted to PD-1 and PD-L1 [16, 17, 18].

In melanoma, Brahmer et al. (2012) [6] reported a response rate of 17% in patients receiving anti-PD-L1, whereas the percentage of responders for anti-PD-1 was around 19–44%, slightly higher than in non-small cell lung cancer, 16–30% [19, 20, 21]. It is worth noting that the most representative effect was a delay or control of the tumor growth, suggesting that adequate combination would probably enhance the antitumor response.

Based on the discrepancy across the populations after treatments, the effort is currently focused on the identification of certain biomarkers associated with the disease evolution [10, 22]. Personalized medicine in order to predict the individual biology of the immune system, represents the optimal approach. In that sense, Teng and coworkers (2015) [10] have suggested that one of the major factors to predict the response for anti-PD-1/PD-L1 therapy, is the expression of the ligand in tumor cells. Thus, patients with PD-L1+ tumors had higher response rate than patients who did not or poorly express this ligand [23], although this does not always rule. Therefore, the major controversy is about the technical assays and criteria used across the different clinical studies to establish the limit between positive and negative PD-L1 expression [4]. In this work, B16-OVA cell line was classified as PD-L1+ by cytometry analysis. Moreover, the anti-PD-L1 administration in mice led to a rapid tumor infiltration characterized by an increment of tumor specific CD8+ (OVA-CD8+) lymphocytes. These infiltrates reached the maximal peak at 8 h post-dosing. This response was dependent on the tumor antibody kinetics, demonstrating that ligand binding and its blockade triggered this infiltrating T-cells immune response. In line with this result, it has also been reported a rapid response for anti-PD-1, early 8 weeks, but associated with a relative low efficacy [24]. Therefore, other factors are able to modulate the final outcome. Hence despite one of the major therapeutic predictors is the expression of PD-L1, even under this situation, clinical efficacy is still low. Thus, some authors propose the presence of TILs together with PD-L1+ expression, as responsible drivers of the adaptive immune resistance; while no TILs, indicate intrinsic induction [10]. However although the current melanoma model could be classified as PD-L1+/TIL+, additional variables play a relevant role in the global therapeutic response due to the presence of non-responders [15].

On the other hand, changes in PBLs kinetics represent an interesting approach to explore the immune response at individual level. In this work, PBLs in blood and OVA-CD8+ in tumor followed similar time kinetics, increasing, decreasing and achieving a peak at 8 h post- antibody dose, yielding rather the predictive peripheral biomarker concept. This finding needs further additional studies, especially due to the expression of PD-L1 in peripheral-blood T cells. However in this line, peripheral lymphocytes have been described as possible biomarkers associated with disease outcome for Ipilimumab [25]. Thus, the overall survival in patients with melanoma has been related to an increase in the absolute lymphocytes count [4].

Taking into account the main findings reported in the literature for anti-PD-1/PD-L1 therapies together with our observations, one of the main question that remains open in the clinical application is the optimization of the therapeutic regimens. The principal characteristic for these biomolecules is a long half-life with the objective to attain the maximum desired effect with reduced number of doses. This is the case of the anti-PD-L1 evaluated by Brahmer and coworkers [16] in a clinical trial involving patients with several types of cancers. The serum half-life in these patients was estimated in approximately 15 days, whereas the receptor occupancy in blood was > 65% at the end of the first cycle with independence of the dose. A similar situation was described for the anti-PD-1 (Nivolumab) with a serum half-life between 12–20 days and a blood sustained receptor occupancy >70% for more than 2 months [26]. In the present work, the activation of the immune system took place very rapidly and just after three administrations the tumor regression or its control could be assumed. This regimen has been also reported by other authors [27]. Now the question should be addressed to investigate adequate dosing schedule or therapeutic schemes as well as possible combinations. Moreover, special attention should be also taken in the pharmacokinetics of mAbs and their immunogenicity displayed after several administrations, even when this characteristic has been diminished with the last full humanized antibodies [28].

Therefore, anti-PD-L1 antitumor effect was supported by the correlation between antibody tumor concentrations and specific infiltrating lymphocytes found in B16-OVA tumor bearing mice. This mechanism was also reflected by the PBL modulation at peripheral blood samples, establishing a possible relationship between these lymphocytes and the anti-PD-L1 kinetics in the target tissue.

## MATERIALS AND METHODS

### Cell line

B16-OVA tumor cell line was cultured in DMEM (GIBCO^®^-Spain) supplemented with 10% Fetal Bovine Serum (FBS; GIBCO^®^-Spain), 1% penicillin/streptomycin (GIBCO^®^-Spain), 1% L-Glutamine (200 mM; Lonza^®^-Spain) and 50 μM β-mercaptoethanol (Sigma Aldrich^®^- Spain). This cell line, provided by Dr. D. Llopiz (CIMA, University of Navarra, Spain) was derived from B16F10 mouse syngeneic melanoma cell line transfected with chicken OVA, which its expression was maintained in presence of 400 μg/mL Geneticin (50 mg/mL, Lonza^®^-Spain).

### *In-vitro* experiments

B16-OVA cell line seeded at a cell density of 1 × 10^6^ cells/well in 12 wells-microtiter plates and incubated at standard conditions, were used to performer the following experiments.

### PD-L1 cell expression

B16-OVA cells were detached with citrate solution 1X and collected at 24, 48 and 72 h after seeding. Cells were labelled with PE anti-mouse CD274 Ab [1:600, v/v] for 10 min. at 4°C, and then, analysed by flow cytometry.

### Impact of anti-PD-L1 treatment in PD-L1 expression

Twenty-four hours after seeding, cells were treated with 5, 25 and 50 μg/mL of anti-PD-L1 [clone 10F.9.G2; bioXCell^®^ - USA] for 4 and 24 h. Immediately after, cells were washed with PBS and refreshed with medium. PD-L1 expression was quantified by flow cytometry at several time points after treatment to evaluate the binding selectivity of the antibody to the ligand, complex internalization and ligand recycling.

### Anti-PD-L1, mechanism binding to PD-L1

B16-OVA cells 24 h after seeding were treated with 25 μg/mL of anti-PD-L1 for 4 h following three different conditions: 1.- cells attached and incubated at 37°C; 2.-cells attached and incubated at 4°C and 3.-cells in suspension incubated at 4°C. After treatment, cells were washed out with PBS and collected to be labelled for PD-L1 expression quantification by flow cytometry.

### Immunohistochemistry for antibody trafficking

B16-OVA cells seeded in 8-chambers culture slide at a cell density of 1 × 10^4^ cells/well at 37°C, were treated with 50 μg/mL of anti-PD-L1 and split into 6 groups to detect the location of antibody by confocal microscopy. Three groups were incubated for 4, 8 and 24 h (continuous exposure) with the treatment and the other three, was only incubated for 4 h and the imaging was captured just at 4 h after antibody removal, 8 and 24 h post-treatment. All cell samples were fixated with 4% PAF, labelled for 1 h at RT with 2.5 μg/mL of Donkey anti-rat 488 Antibody (Ref. A21208; Life Technology- USA] and 10 μg/mL of DAPI [Ref. 40009; Biotium- Spain] for immunohistochemistry analysis and microscopy imaging.

### *In-vivo* experiments

#### Pharmacodynamics

##### Influence of the initial tumor size in the antitumor effect of anti-PD-L1

Forty eight female C57BL/6 mice (Harlam lab. Inc., Barcelona- Spain) weighting 20–25 g were subcutaneously inoculated with 5 × 10^5^ cells/100 μl PBS in the right flank of the mice and randomly divided into four groups (*n* = 12 mice/group): 1.-Control; 2.-Small Tumor Size (STS); 3.-Medium Tumor Size (MTS) and 4.-Large Tumor Size (LTS). These groups were according to the day of treatment, consisting in 100 μg anti-PD-L1/mouse i.v. Q3Dx4 administrations (one cycle). For STS, mAb was administered at day 3 after tumor cells inoculation, MTS at day 7 and LTS at day 11. Tumor size was measured every two-three days until the end of experiment. The end point criteria for each animal were established following the protocol approved by the Ethic Committee of University of Navarra (ref: 046-14).

#### Pharmacokinetics

##### A.- Time profiles of anti-PD-L1 concentration in serum and tumor biomarkers

Twenty seven B16-OVA tumor bearing mice were randomly divided into two groups: 1.- Control (*n* = 6) and 2.-treated (*n* = 21). Treated animals received a single i.v. administration of 100 μg anti-PD-L1 mAb at day 7 after tumor cells inoculation. At different time points between 10 min and 72 h after antibody administration, groups of 3 animals were sacrificed to collect tumor and blood samples. Anti-PD-L1 concentrations were measured by ELISA in serum. For biomarkers, tumor samples were prepared in small pieces and digested in 5 mL collagenase/DNase [10:1; Roche^®^- France] for 5 min at 37°C and afterwards, 50 μL of EDTA was added. Tumor cells dissociation was completed by centrifugation at 4°C, 2000 rpm for 10 min. Then, cells were lysed with 1 mL of ACK lysis buffer [1X; Lonza^®^- USA]. This reaction was stopped mixing with 200 μL of PBS. Samples were transferred to 96-wells microtiter plates, centrifuged and washed twice with PBS. Cells samples were 10 min incubated in darkness with several labelling antibodies, iTAg™ MHC Class I Murine Tetramer – SA-PE [1:100, v/v; Ref. T03000GE; Immunomics-USA] and after washing with PBS, cells were incubated with a mixture of (FITC)-conjugated anti-mouse CD8α [1:200, v/v; Ref. 10075; BioLegend^®^-USA] and APC anti-mouse CD279 [1:500, v/v; PD-1, Ref. 10911; BioLegend^®^-USA]. These cells samples were washed by centrifugation and maintained at 4°C in PBS until analysis by flow cytometry. Biomarkers in control mice were also measured following the same protocol at two times 0 and 72 h.

##### B) Anti-PD-L1 tumor concentrations

Following the protocol described above, twenty one B16-OVA tumor bearing mice were treated with a single i.v. administration of 100 μg anti-PD-L1 mAb/mouse at day 7 after tumor cells inoculation. At the same time points that those selected to quantify serum levels of mAb, groups of 3 mice were sacrificed to collect the tumor. This tissue was immediately and mechanically homogenized in PBS (0.1 g tissue/mL). Antibody levels in the tumor tissue solution were measured by ELISA. The analytical method was validated for linearity and accuracy. Standard curve in serum was linear in the range 75–1.2 μg/mL, whereas in tumor was from 3.3 μg/mL until 104.2 ng/mL. Accuracy was > 95% for both curves.

### Statistical analysis

Data were expressed as mean ± SD or MSE. The statistical analysis was performed using a two-way ANOVA for the comparison across groups, followed by Bonferroni test to compare two by two the groups. The significance level was set at *P* < 0.05. Graphs were generated with GraphPad Prism software 5.0.
